# Field Experiments on Health and the Built Environment in Urban Settings: A Scoping Review

**DOI:** 10.1007/s40572-026-00539-5

**Published:** 2026-05-19

**Authors:** Alexandra Bretones Diaz, Samuel Nello-Deakin, Jordi Honey-Rosés

**Affiliations:** 1 Departament de Geografia, Universitat Autònoma de Barcelona, Bellaterra, Spain; 2 Institut de Ciència i Tecnologia Ambientals, Universitat Autònoma de Barcelona, Bellaterra, Spain

**Keywords:** Built environment, Field experiments, Health, Tactical urbanism, Environmental exposure

## Abstract

**Purpose of Review:**

Science has long examined how the built environment impacts health and wellbeing, yet most research has relied on observational and longitudinal studies. Much less attention has been paid to field experiments as a method to understand how environmental conditions may impact health. Although formal field experiments are common in public health, environmental psychology and related fields, they remain rare in urban planning and design despite their potential to inform how urban transformations may improve health and wellbeing. This scoping review examines how field experiments have been used to assess the health and wellbeing impacts of the built environment.

**Recent Findings:**

Following an extensive keyword search, we identified studies published between 2015 and 2025 that employed a field experimental design in an urban or peri-urban context and directly assessed health-related outcomes associated with built environment characteristics or interventions. A total of 26 studies met the inclusion criteria and were analysed according to their topical focus, methodological design, assessed health outcomes, and reported challenges and limitations. Most studies focused on the health and wellbeing impacts of exposure to urban green features, accounting for over two thirds of reviewed studies. Mental health and wellbeing outcomes were assessed more frequently than physical health outcomes, often through a combination of self-reported and objective physiological measures. Only a limited number of studies involved direct interventions or transformations of the built environment, while most compared existing environmental conditions.

**Summary:**

The limited number of field experiments addressing issues beyond urban green features suggests there is considerable opportunity to expand the use of field experiments to assess the health impacts of other types of built environment interventions, particularly in the context of the growing popularity of tactical urbanism and temporary urban transformations.

## Introduction

A longstanding scientific puzzle concerns establishing a causal relationship between the built environment and our health and wellbeing [1]. How does the built environment impact our health, in terms of physical, mental and social wellbeing? What are the features of a healthier city and what causal evidence do we have that specific urban designs improve our wellbeing? Architects and planners have long argued that strategic design interventions can build healthier communities [2], and yet the evidence base to support many of these claims is tenuous.

Establishing causality is challenging because we confront fundamental research design problems such as selection bias. Individuals that live in particular cities or neighbourhoods are also likely to share common values and belief systems that will also change their habits, and therefore health [3]. So how can researchers isolate the causal relationship between built environment and health outcomes?

For decades, most research on these questions has relied on observational study designs, including cross-sectional, case-control, ecological and longitudinal studies, often based on health surveys and regression models to block the effect of confounding variables and bring correlation estimates closer to a causal estimate [4–6]. Longitudinal studies, in particular, offer advantages in capturing changes over time and strengthening causal inference compared to cross-sectional approaches. However, observational studies remain limited in their ability to establish causality due to issues such as selection bias, residual confounding, and self-selection into neighbourhoods. As a result, while observational studies provide valuable insights into correlations and potential pathways, they are often insufficient to directly inform decision-making on urban interventions aimed at improving health and wellbeing.

Another approach has been to use natural experiments, which exploit natural variations in urban conditions to tease out health impacts of different living conditions. Research with natural experiments has become increasingly common, as evidenced by multiple literature reviews on the topic [7–10]. However, natural experiments rely on real-world settings, making it difficult to control for potential sources of bias and dynamic interplays between confounding factors [7, 11]. As a result, establishing rigorous and conclusive causal evidence remains difficult.

Of the various research designs used to examine the relationship between our health and the built environment, the design that has received the least attention has been field experiments. Field experiments are randomised studies conducted in real-world settings [12], or data collection strategies that “employ manipulation and random assignment to investigate preferences and behaviors in naturally occurring contexts” [13]. In practice, field experiments are typically characterised by several key design features, including: (i) the presence of a treatment or intervention (e.g. a modification of the built environment or exposure to different environmental conditions); (ii) the assignment of participants to different conditions, ideally through randomization; (iii) implementation in real-world contexts rather than controlled laboratory settings; and (iv) the measurement of behavioural, physiological, or self-reported outcomes. In some cases, field experiments entail active manipulation of the built environment to assess impacts on health, while in other cases researchers randomize participants into groups which are exposed to different conditions within urban or peri-urban built environments, including variations in green and blue infrastructure. Field experiments offer a distinct methodological contribution by enabling stronger causal inference than observational studies, while maintaining high internal validity. While experimental designs are common in other fields, they are rare in urban design, planning and architecture, because these fields do not have a strong tradition in formal experimentation [14, 15]. The conventional wisdom among urban planners and designers is that formal experiments with randomization and comparison groups are not possible in urban planning [16]. And yet field experiments have the potential to contribute valuable scientific evidence on the relationship between health and the built environment.

Furthermore, the rise in tactical urbanism interventions make field experiments more feasible. Tactical urbanism is a low-cost, temporary, and scalable approach to improving urban spaces through short-term interventions that test and demonstrate long-term planning solutions [17, 18].

To this end, we conducted a structured search for relevant studies and synthesised evidence on their focus areas, methodological design, statistical approaches, health outcomes, and reported challenges and limitations. By doing so, this scoping review provides an overview of how field experiments are currently applied to assess the health impacts of the built environment, as well as the opportunities and constraints associated with their use.

## Methods

Scoping reviews are appropriate to identify dominant research themes and methodological approaches, and highlight evidence gaps, rather than to synthesise effect sizes or formally assess study quality [19]. Our approach to undertaking the review was inspired by the PRISMA-ScR (Preferred Reporting Items for Systematic Reviews and Meta-Analyses extension for Scoping Reviews) guidelines [20], but given the aspiration of CEHR articles to provide succinct readable summary articles, we consciously do not adhere to the full list of PRISMA reporting items.

We carried out a systematic search in the Scopus database, selected for its broad multidisciplinary coverage of urban studies, public health, environmental health, and planning research. Our final search query was TITLE-ABS-KEY (“field experiment” AND “health” AND “urban” OR “built environment”). Searches were limited to peer-reviewed journal articles published in English language between 2015 and 2025. We performed the search on 25 September 2025, yielding an initial total of 166 records. Our search strategy was intentionally conservative to maximise specificity towards field experiment studies, acknowledging that the term “field experiment” is not always consistently used across disciplines.

After this initial step, we undertook a multi-stage screening and selection process to determine eligibility for inclusion in the scoping review. Studies were included if they met all the following criteria:

i) The study must self-define as a “field experiment” and take place in a real-world environment; accordingly, studies using laboratory experiments, stated preference experiments, simulations, modelling exercises, or purely observational designs were excluded.
ii) The study must be conducted in an urban or peri-urban context. Studies conducted exclusively in rural or non-urban environments were excluded, as were studies comparing urban and non-urban or rural settings (e.g. urban versus rural spaces or agricultural land uses).
iii) The study must evaluate a built environment intervention or compare the environmental characteristics of different urban/peri-urban environments. These may include but are not limited to aspects related to green or blue infrastructure, public spaces, streets, transport infrastructure, or other urban facilities.
iv) The study needs to directly assess some type of human health outcome, including physical health, mental health, mood, wellbeing, or self-reported health status. Studies focusing exclusively on indirect or proxy outcomes such as environmental exposures (e.g. air pollution levels, noise, temperature, or soil quality) or physical activity were therefore excluded.

Based on a preliminary screening of abstracts, publications meeting the established inclusion criteria were selected for full-text screening and review. Any uncertainties regarding eligibility were resolved through discussion and consensus by the three researchers AB, SN and JH.

For each study, we extracted information on study location, type of environmental intervention or characteristic under study, experimental design (e.g. use of control conditions, randomization), and measured health outcomes. Health outcomes were classified into two broad categories, either as physical (e.g. blood pressure, heart rate, cortisol, physiological stress markers) or mental (e.g. mood, perceived restorativeness, stress, affective responses).

## Findings

Based on the criteria described in the methods section, we selected a total of 26 studies from the initial 166 identified records. The geographic setting of existing research is heavily slanted towards countries in Asia (15 studies) and China in particular (7 studies), while 6 studies were conducted in Europe and 4 in North America. The results of our scoping review are organized by the area of focus, methodological design, health impacts, statistical analysis, and challenges and limitations, and are summarized in Table 1.

**Table 1:** Summary of studies adopting experimental approaches to understanding the relationship between health and the built environment

Author/Date	Country	Category	Built environment focus	Intervention	*N*	Randomization	Control Conditions	Objective measures	Self-reported measures	Physiological/physical health outcomes	Psychological/mental health outcomes
Chou et al. 2016 [21]	Taiwan	Environmental exposure	Relationships between urban open spaces and human health	N	565	N	N	N	Y	N	Y
Dehove et al. 2024 [22]	Austria	Public Space Transformation	Parklet urban art intervention	Y	130	Y	Y	Y	Y	Y	Y
Elsadek et al. 2019 [23]	China	Environmental exposure	Influence of roadside trees on stress relief	N	364	N	N	N	Y	N	Y
Gao & Zhu 2025 [24]	Singapore	Environmental exposure	Outdoor lighting of greenery	N	428	N	Y	N	Y	N	Y
Gao & Zhu 2025 [25]	China	Environmental exposure	Pathways in urban parks in winter	N	360	N	N	N	Y	N	Y
Graif et al. 2016 [26]	United States	Policy Experiment	Neighbourhood effects on mental health through housing voucher experiment	Y	3000	Y	Y	N	Y	N	Y
Guéguen & Stefan 2016 [27]	France	Environmental exposure	Testing altruism and pro-social behaviour and mood in green spaces	Y	640	Y	Y	Y	Y	N	Y
Honey-Rosés & Zapata 2023 [28]	Canada	Public Space Transformation	Effects of increasing pedestrian density	Y	506	Y	Y	N	Y	N	Y
Kabisch et al. 2021 [29]	Germany	Environmental exposure	Monitoring effects of exposure to urban green spaces among older adults	N	33	Y	Y	Y	Y	Y	Y
Kim et al. 2022 [30]	Korea	Environmental exposure	Effects of Green Space in an Apartment Complex on the Environmental Cognition and Stress Response of female homemakers	N	16	Y	Y	N	Y	N	Y
Korpilo et al. 2024 [31]	Finland	Environmental exposure	Urban natural environments and soundscapes	N	45	Y	Y	Y	Y	Y	Y
Lanki et al. 2017 [32]	Finland	Environmental exposure	Effects of visits to urban green environments on cardiovascular physiology in women	N	40	N	N	Y	Y	Y	N
Li D et al. 2023 [33]	USA	Environmental exposure	Contextual and environmental factors influencing health	N	39	N	N	Y	N	Y	N
Li, Y et al. 2023 [34]	China	Environmental exposure	Restorative benefits of non-virtual environments among young adults	N	39	N	N	Y	Y	Y	Y
Luo et al. 2023 [35]	Japan	Environmental exposure	Restorative effect of urban blue spaces among students	N	41	Y	Y	N	Y	N	Y
Luo et al. 2024 [36]	China	Environmental exposure	Residential outdoor spaces (elderly population)	N	69	N	N	Y	Y	Y	Y
MacDonald et al. 2024 [37]	USA	Public Space Transformation	Abandoned housing remediation	Y	258	Y	Y	Y	N	Y	N
Raman et al. 2021 [38]	Malaysia	Environmental exposure	Effects of different natural environments on health and psychological wellbeing among working adults	N	80	Y	Y	N	Y	N	Y
Saadi et al. 2020 [39]	Israel	Environmental exposure	Exposure to urban environments with intra-ethnic diversity	N	72	N	N	Y	Y	Y	Y
Schlinkert et al. 2020 [40]	The Netherlands	Public Space Transformation	Food truck intervention to promote healthy eating	Y	NR	N	N	Y	N	N	N
Souter-Brown et al. 2021 [41]	New Zealand	Environmental exposure	Exposure to sensory garden in the workplace	N	164	Y	Y	Y	Y	Y	Y
Xu et al. 2024 [42]	China	Environmental exposure	Urban pocket parks	N	96	Y	Y	Y	Y	Y	Y
Yin et al. 2023 [43]	Taiwan	Environmental exposure	Effect of blue space exposure to physiological and psychological response among young adults	N	20	Y	N	Y	Y	Y	Y
Yuan et al. 2023 [44]	China	Environmental exposure	Women physiological wellbeing in relation to the environment (natural vs. built)	N	10	Y	N	Y	Y	Y	Y
Zhang et al. 2025 [45]	China	Environmental exposure	Outdoor sites with greenery in winter (elderly population)	N	345	Y	N	Y	Y	Y	Y
Zhao et al. 2025 [46]	China	Environmental exposure	Linear landscape features	N	200	Y	Y	Y	Y	Y	Y

### Areas of Focus

A key finding is that two thirds of reviewed studies (18 of 26) focus on assessing the health and wellbeing impacts of green elements (e.g. urban parks, tree coverage, green corridors) within an urban context, with two additional studies [35, 43] examining urban blue spaces. These studies compare the health impacts associated with different or contrasting built environments. We categorize these studies as *environmental exposure* (Table 1), and this was the dominant study type. Experimental studies that manipulated public space are rare and limited to singular studies looking at questions about outdoor lighting [47], abandoned housing [37], pedestrian density [28], quality of residential outdoor spaces [36], and neighbourhood poverty and disadvantage [26].

### Methodological Design

While all studies were considered field experiments, the level to which their methodological design conformed to the characteristics of an ideal-type field experiment (i.e. including randomization, a clearly defined treatment, and the use of control conditions) varied. In this respect, an important finding is that only 6 studies developed an intervention to alter the built environment. In the remaining cases, studies compared pre-existing urban conditions.

In 14 cases, the study design explicitly included a control site or control condition to compare with a “treatment” condition, while the remaining studies compared different environmental conditions but without defining one of them as a control condition. Studies generally rely on either a within-subject (15 cases) or between-subject (13 cases) experimental design, with only 3 studies incorporating both types of design.

Participant sample sizes vary widely between studies, ranging from 10 [44] to over 3,000 [26]. Often studies focused on how specific populations (students, elderly, women, employees) were impacted by conditions in the built environment yet given the different characteristics of the studies it is difficult to extract common guidelines on this front.

### Health Outcomes

We found that most studies combine both objective and self-reported health measurements: 17 out of 26 studies assess objective health outcomes through objective sensor or tracker data, while 23 studies include self-reported health or wellbeing outcomes, generally using a standardised questionnaire or survey.

This choice of method generally bears a relationship with the type of health outcomes being assessed: physical health outcomes (assessed in 15 studies) are typically evaluated using objective data from body sensors, while mental health or wellbeing outcomes (assessed in 22 studies) are mostly evaluated using self-reported questionnaires. The type of physiological responses studied varied from study to study depending on its exact focus and design, with some of the most common measures including blood and heart rate pressure, and electrodermal activity. Less common measures include eye tracking, electroencephalograms, or saliva samples to measure cortisol levels. While studies assessing self-reported health or wellbeing measures use a variety of scales and approaches, Hartig’s Perceived Restorativeness Scale (PRS) stands out for its use in multiple reviewed studies [48].

### Statistical Analysis

A wide range of quantitative analytical techniques were used to test the significance of health outcomes across reviewed studies. Most studies relied on comparative statistical tests to examine differences between experimental conditions, most commonly ANOVA and t-tests, including paired, independent-sample, and non-parametric equivalents (e.g. Mann–Whitney U and Wilcoxon signed-rank tests) [30, 33–35, 38, 44]. Several studies complemented these analyses with regression-based approaches, including linear regression, mixed-effects models, generalized additive mixed models (GAMM), or logistic regression [28, 43], and difference-in-differences designs [37] when control groups or repeated measures were available. A smaller number of studies applied multivariate or advanced analytical techniques, such as structural equation modelling [45], mediation analysis [24], random forest models [25], and non-metric multidimensional scaling [35].

### Challenges and Limitations

Among commonly mentioned limitations in the reviewed studies, multiple studies highlight the short time frame of field experiments as a key shortcoming: typically, most field experiments are carried out on a single day and only measure the momentary health outcomes of environmental exposure. In other word, analytical strategies were predominantly short-term and focused on detecting immediate changes in health outcomes following environmental exposure or interventions. Beyond making it impossible to account for the potential effects of seasonality and weather, this ignores the long-term health effects of built environment characteristics, which may be more significant in many cases. Admittedly, such long-term effects may be more amenable to study using natural experiment designs rather than field experiments, but there is arguably an opportunity to further explore the potential of long-term field experiments in cases where they may be feasible [26, 37]. In this respect, relying on externally gathered health impact data [37], as opposed to data collected directly by the researchers, may offer a way to assess the longer-term impacts of field experiments.

Other methodological challenges mentioned in multiple studies included difficulties in identifying appropriate control conditions, heterogeneity in exposure protocols (e.g., walking speed, route length), and the lack of objective physiological measurements in some studies, with reliance on self-reported outcomes. Even where physiological data were collected, several authors noted that additional measures (e.g., cortisol, skin conductance, or eye-tracking) could have strengthened causal inference. Likewise, several studies also reported limited exposure durations (e.g., 10–30 min), small sample sizes, and a focus on specific population groups (such as young adults or healthy participants), restricting generalisability.

## Discussion & Conclusion

This review of field experiments on questions about urban health identifies key trends and prospects. Perhaps our main finding is the predominance of studies focusing on the health impacts of exposure to green/blue spaces, particularly on wellbeing and restorativeness, which account for over two thirds of all reviewed studies. Since environmental psychologists have a strong tradition of working with experimental methods, this is perhaps to be expected. Nevertheless, the preponderance of studies focusing on towards green/blue spaces suggests there is a missed opportunity to apply field experiments to other types of built environment interventions and features. Our review has uncovered a highly creative yet small group of studies that have attempted to use an experimental framework to study innovative built environment interventions. For instance, the work by Dehove et al. [22] on the aesthetic designs of streetscape, or the work by Schlinkert et al. [40] to learn about how the installation of temporary food trucks might change consumer eating habits. These experimental papers have their respective research design challenges, but they also show that experimental methods can be adopted to a different family of questions outside the issue of exposure to greenspace and blue space. Furthermore, given the current wave of interest in tactical urbanism [18, 49] and street experimentation [50, 51], field experiments offer a valuable way of testing the impacts of such interventions, both in terms of wellbeing and physical health/activity outcomes. For instance, the research by MacDonald and colleagues [37] on abandoned housing remediation offers a creative example of how field experiments can be productively applied to topics other than natural environment restorativeness.

A second key finding is that most studies (20 of 26) do not actively transform the built environment or public space but merely compare existing environments. It is understandably difficult for researchers to change the built environment, especially if they are working alone and without the collaboration of city officials. Therefore field experiments need close collaboration with city leaders to develop policy experiments such as the work done by MacDonald et al. [37] which assessed real changes implemented by the city, or the work by Graif et al. [26] which exploited the Move to Opportunity experiment developed by the US Federal Housing Authority.

The limited use of field experiments to study health impacts of the built environment might also be explained by the challenges in fostering collaboration between urban and health researchers [52, 53]. This research is dominated by researchers from health and environmental psychology, but urban researchers from disciplines such as geography, urban design and planning are perhaps more likely to generate theoretically valuable and policy-relevant questions to test through a field experiment. Nevertheless, they are unlikely to have the technical knowledge, equipment or expertise to measure physical activity and health indicators.

Social interventions, such as social programs, are notoriously much more well suited to field experiments than built environment interventions, because social programs can be more easily randomized over individuals [13] while place based interventions are more difficult to randomize. Field experiments also have their limitations. While they may have high internal validity, they may have less relevance to other sites, and therefore weaker external validity or construct validity. Field experiments are also reductionist, focusing narrowly on a set of variables that may or may not target the right questions needing answers.

Furthermore, we would like to acknowledge that our scoping review may have omitted studies which use field experiments to test the health impact of specific built environment features on health, but do not explicitly use this terminology. An example of this are field experiments testing the impacts of infrastructural interventions on traffic safety [54, 55], which arguably may be considered as a type of health outcome. Likewise, our search only extends to English-language articles captured in the Scopus database for the 2015–2025 period.

Despite these challenges, experimental methods hold an untapped potential to test the health and wellbeing impacts of urban conditions and transformations. In particular, there is potential to test “soft” built environment interventions such with temporary or mobile elements. Currently, the use of field experiments to study the health impacts of our built environment remains limited and specialized among researchers developing exposure studies on the impact of green and blue spaces on our wellbeing. Only a handful of studies have begun to use experimental methods on other interventions and wellbeing outcomes.

In conclusion, field experiments offer a valuable yet underused opportunity to strengthen causal evidence on the health impacts of the built environment. While existing studies provide consistent evidence of short-term wellbeing benefits associated with exposure to green and blue urban environments, far fewer experiments have examined other types of built environment interventions, particularly those involving active transformations of public space. Expanding the use of field experiments beyond environmental exposure studies, and combining them with longer-term designs, policy experiments, and interdisciplinary collaboration, could substantially strengthen causal evidence in urban health research. As cities increasingly experiment with temporary, tactical, and adaptive interventions, field experiments offer a timely and valuable tool to better understand how urban change can support healthier and more equitable cities.

## Data Availability

No datasets were generated or analysed during the current study.
